# Feasibility and Acceptability of ‘VitaVillage’: A Serious Game for Nutrition Education

**DOI:** 10.3390/nu14010189

**Published:** 2021-12-31

**Authors:** Nienke M. de Vlieger, Lachlan Sainsbury, Shamus P. Smith, Nicholas Riley, Andrew Miller, Clare E. Collins, Tamara Bucher

**Affiliations:** 1 College of Engineering, Science and Environment, School of Environmental and Life Sciences, The University of Newcastle, Callaghan, NSW 2308, Australia; lachie.sainsbury@gmail.com (L.S.); shamus.smith@newcastle.edu.au (S.P.S.); Tamara.Bucher@newcastle.edu.au (T.B.); 2 Priority Research Centre in Physical Activity and Nutrition, The University of Newcastle, Callaghan, NSW 2308, Australia; Nicholas.riley@newcastle.edu.au (N.R.); clare.collins@newcastle.edu.au (C.E.C.); 3 College of Human and Social Futures, The University of Newcastle, Callaghan, NSW 2308, Australia; Andrew.Miller@newcastle.edu.au; 4 Teachers and Teaching Research Centre, School of Education, Faculty of Education and Arts, The University of Newcastle, Callaghan, NSW 2308, Australia; 5 College of Health, Medicine and Wellbeing, School of Health Sciences, The University of Newcastle, Callaghan, NSW 2308, Australia

**Keywords:** gamification, serious games, children, nutrition, education, Australia

## Abstract

Computer games have previously been used to improve nutrition knowledge in children. This paper describes the acceptability and feasibility of a serious game, “VitaVillage”, for improving child nutrition knowledge. VitaVillage is a farming-style game in which the player undertakes quests and completes questions aimed at increasing several aspects of nutrition and healthy eating knowledge. Children aged 9–12 years in two primary schools (control vs. intervention) completed a nutrition knowledge questionnaire at baseline (T1) and after 1 week (T2). Participants at the intervention school (*n* = 75) played VitaVillage for 20 minutes on two occasions. Control participants (*n* = 94) received no nutrition education. Likeability question scores and written feedback from intervention participants was reported qualitatively. Paired sample *t*-tests were used to compare T1 and T2 nutrition knowledge changes between control and intervention participants. Engagement with VitaVillage improved children’s overall nutrition knowledge (Mean increase of 2.25 points between T1 and T2, Standard Deviation (SD) 6.31, *p* = 0.035) compared to controls. The game was liked overall (mean score 77 (SD 24.6) on scale of 0–100) and positive feedback was given. Results indicate that VitaVillage has the potential to be successful as a nutrition education tool. In the future, VitaVillage’s content and gameplay will be revised, extended and evaluated for its long-term impact on eating behaviour and knowledge changes.

## 1. Introduction

The dietary patterns of many Australian children are not optimal with high intakes of energy-dense, nutrition-poor (EDNP) foods, and low intakes of fruit and vegetables [1]. To ensure children’s diets are nutritionally adequate and support optimal health outcomes, it is important to provide support to develop healthy eating habits [2]. 

Parents have a major influence on what their child consumes and learns about food at home [3,4]. Previous literature has found an association between low parental education levels and less comprehensive nutrition knowledge of their children, and therefore not all children will be taught about healthy eating behaviours at home [5,6]. However, children also spend a large portion of their time in school and are influenced by their teachers, peers and their school’s curriculum [7]. This is of particular interest for nutritional education opportunities, as schools have the benefit of reaching almost all children. Unfortunately, nutrition education is not a mandatory subject in many primary schools and often makes way for core subjects such as English and Mathematics [8,9]. In a survey among Australian primary school teachers, lack of confidence and sufficient resources were named as additional barriers to teaching nutrition in class [9].

Serious games are increasing in popularity and are a potential method for teaching children about a range of health topics and promoting healthy behaviours, including those related to nutrition [10,11,12,13,14]. It has been suggested that serious games may be a good alternative to traditional teaching [15,16], which is not surprising considering the popularity of video games among children [17] and their preference for Information and Communication Technologies (ICTs) over printed materials [18,19]. Several studies identified that educational nutrition games were superior in improving children’s nutrition knowledge compared to traditional learning [14,20,21], while a recent review on nutrition education and games found that 21 out of 22 studies reported positive changes in food intake, such as an increase in fruit and vegetable consumption or a reduction in sugar intake [22]. 

The integration of (educational) games into the classroom has become increasingly popular among teachers as they believe it to be an innovative method of engaging and motivating students and can improve students’ skills and knowledge [23]. Furthermore, as teachers have indicated they lack the time and confidence for teaching nutrition in class [9], access to an evidenced based game designed by nutrition experts might be a valuable resource for teachers.

A multi-disciplinary approach was used to develop an educational, or serious-, game (VitaVillage) that can be used in Australian classrooms to teach nutrition. The aim of the current study was to investigate the feasibility and acceptability of VitaVillage as a nutrition education tool in primary schools. The likeability of the game was tested using a survey to collect feedback about the use and features of the game. In addition, the effectiveness of the game in increasing children’s short-term nutrition knowledge was explored. 

## 2. Materials and Methods

### 2.1. Game Development

A team consisting of nutritionists, dietitians, computer scientists and education experts from the University of Newcastle (Australia), collaborated in developing VitaVillage. Such a collaboration is vital for the development of a serious game that is multidisciplinary in nature [24]. When developing the questions in the game, each question within each nutrition knowledge category was designed to cover the most important/useful nutrition information, as determined by the nutritionists. Additional focus was placed on areas of nutrition knowledge that were found to be gaps during development of the nutrition knowledge questionnaire used in this study. 

To ensure VitaVillage is meaningful and offers a valuable learning experience, a theoretical framework that integrates pedagogy, play and fidelity [24] was used for the development of VitaVillage. According to this framework, pedagogy, play and fidelity must be balanced in an educational game. The learning within games must have a pedagogical underpinning and for the development of VitaVillage, the Quality Teaching Framework, which is used in Australian New South Wales schools, was applied [25]. Ensuring a good balance between pedagogy and play is crucial to the effectiveness of a game [26]. Following the serious game framework [24], elements of engagement, flow and immersion were incorporated. To increase the fun elements in VitaVillage, the player controlled character was positioned as a third-person avatar within the VitaVillage world and supported by an overall game narrative. Lastly, the third element in the theoretical framework is fidelity [24]. This refers to how much the games looks, feels and sounds like the real world (physical fidelity) in addition to how the game acts like the real world in its responses to players’ actions (functional fidelity) [27]. VitaVillage incorporated functional fidelity by simulating the life of a farmer and a realistic representation of the production of food. 

**Figure 1:**
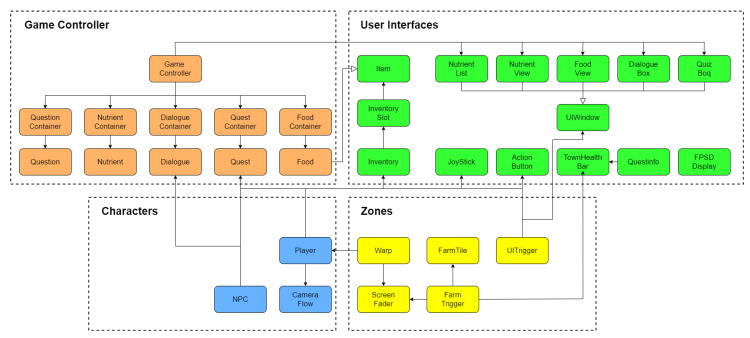
VitaVillage high-level class diagram.

VitaVillage was developed and deployed using the game engine Unity3D (unity.com) which provides built-in game functionality and allows for deployment to many different technology platforms. All game assets were sourced from the Liberated Pixel Cup (LPC) [28]. Four scripting modules were required for this project and defined as: Game Controller, User Interfaces, Characters and Zones (see Figure 1).

The ‘Game Controller’ module was responsible for initialising required data elements from game configuration Extensible Markup Language (XML files). The ‘User Interfaces’ module was responsible for displaying all interactive elements and processing the events triggered by player interaction with these elements. The ‘Characters’ module was responsible for handling the functionality of each in-game agent, classified as either a player or a Non-Player Character (NPC). Lastly, the scripting module ‘Zones’ controlled the firing of events when the player interacted with in-game objects. All educational content in the game was designed in collaboration between the nutrition experts and education experts on the project team. Throughout the game development, content and game features were iteratively developed using an Agile software engineering approach. A large repository of game content was developed, from which a selection was chosen for the current version of VitaVillage.

VitaVillage can be played on a device running the Android operating system and can be downloaded onto a smartphone or tablet via a web link to the application file.

### 2.2. Questing in VitaVillage

**Figure 2:**
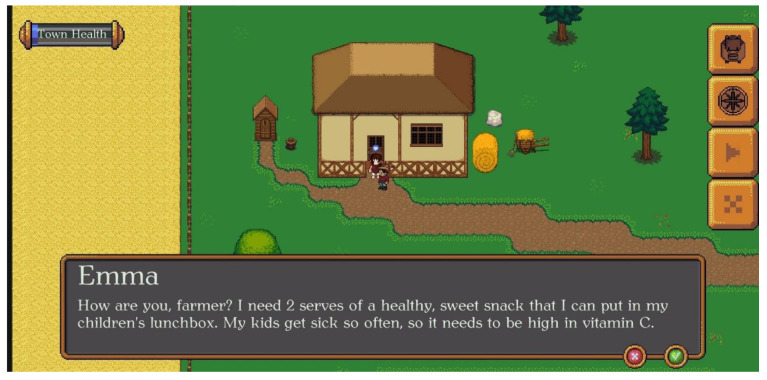
A villager asks the farmer to help them find a snack food high in vitamin C. The player will have to visit the village’s grocery shop. The village shop’s inventory is sorted by vitamins, minerals and dietary fibre. After clicking on a nutrient, a list of associated foods with basic information appears (e.g., Vitamin C lists strawberries, broccoli, potatoes and tomatoes) (see Figure 3).

**Figure 3:**
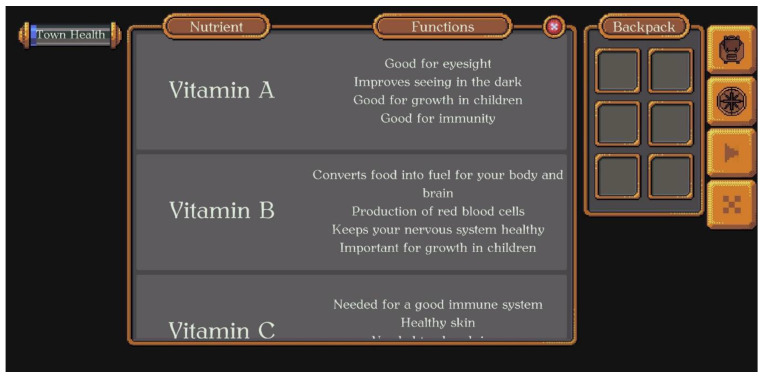
After choosing a nutrient in the shop list, the player can choose to ‘buy’ seeds for the foods that need to be grown, or information appears on where they have to go to acquire the food (e.g., milk is the product under ‘calcium’, for which the player has to milk the cow at their farm.

**Figure 4:**
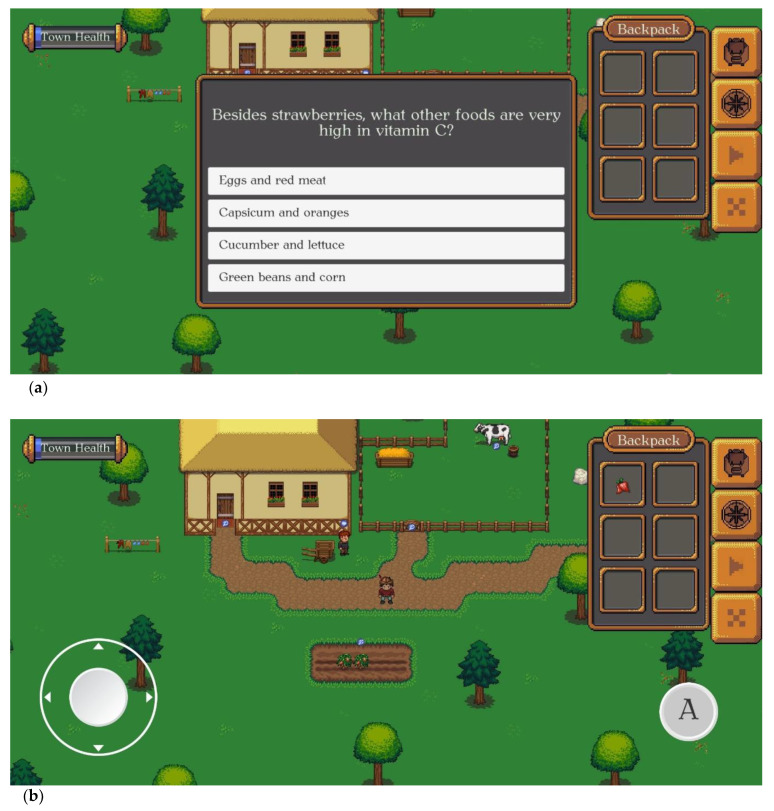
(**a**) A quiz question pops up after planting the seeds. The crop will grow to full size by correctly answering three questions. (**b**) After three correct responses, the crop can be harvested and delivered to the NPC villager in return for points towards the ‘town health’ (see top left-hand corner).

VitaVillage is the name of the town this adventure game takes place in. The player is a farmer in the town who has to make the town as healthy as possible. This can be achieved by completing nutrition-related quests and responding to quiz questions that the NPC villagers pose to the player/farmer. When the farmer approaches a NPC villager, the villager either asks one question about nutrition, which if answered correctly, earns points towards the ‘town health’, or the NPC requests that the farmer helps them grow/retrieve a product that will help them with their health. For example, a villager may ask the farmer to ‘help them get a sweet snack food high in vitamin C’ (see Figure 2). These quests require the player to visit VitaVillage’s shop, where the player will find a list of food items high in that nutrient (see Figure 3). Once the player finds the food that best fits the quest, they can ‘buy’ seeds and put them in their backpack (see Figure 3). The player can grow the seeds into food at their farm (see Figure 4). The farmer grows the seeds into food by correctly answering quiz questions on the current nutrient of interest. If a question is answered incorrectly, the player will get feedback and can try another question. Once three questions are answered correctly, the food can be harvested and taken back to the NPC villager who requested it. Upon completion, the ‘town health’ score bar will increase, and another quest can be started.

When a player plays for the first time, they will have to attend the town’s school, where the teacher briefly explains what nutrition is and asks them a few questions regarding nutrition, nutrients and foods (these do not count towards the town’s health score). Once the school component finishes, the player can start their first quest to improve the town’s health.

The current version of the game contained questions about macro- and micro-nutrients, size and number of recommended daily serves of fruits and vegetables (as set by the Australian Guide to Healthy Eating (AGHE) [28]), balanced meals, food safety and food sources.

### 2.3. Participants and Study Design

Students in year 5 and 6 classes (aged 9–12 years) were recruited from two local schools in Newcastle, New South Wales (NSW), Australia. Principals, teachers and the student’s parents (or carers) gave informed consent for participation before commencing the study. The students themselves also assented to the collection of their data.

One school was randomly allocated to host the control group and the other school to host the intervention group. It was decided to randomise per school, as opposed to per class or even per student, to avoid students in the intervention group talking about VitaVillage to those who were in the control group. 

**Figure 5:**
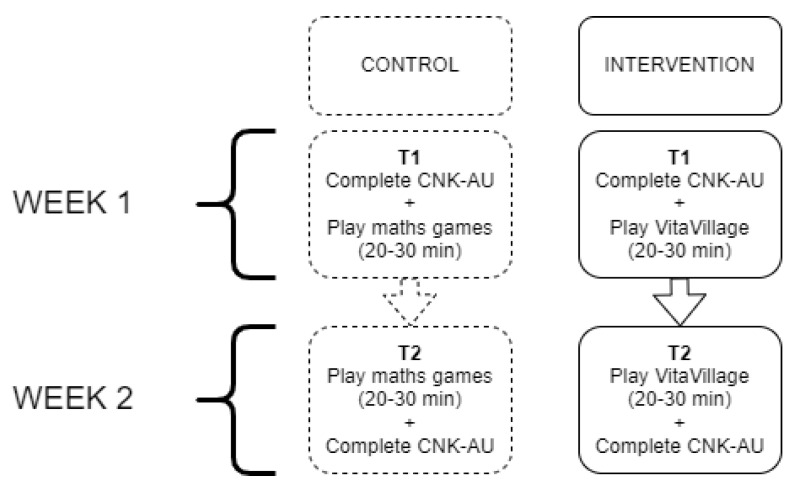
Flowchart of study design. CNK-AU: the Child Nutrition Knowledge Questionnaire-Australian version.

At baseline (T1), both groups completed a nutrition knowledge questionnaire on an Android tablet. Immediately after, the control group played mathematics games and the intervention group participants played VitaVillage for approximately 20 minutes. Exactly one week later (T2) the researchers returned to the schools, where the control participants played mathematics games and the intervention group played VitaVillage again for 20 minutes. Participants in both groups then completing the nutrition knowledge questionnaire for the second time. See Figure 5 for a flowchart depicting the study design. The control group students were given the opportunity to play VitaVillage at the completion of the study. As a thank-you for their participation, all children received a novelty eraser or pencil after the study.

All procedures were approved by the University of Newcastle Human Research Ethics Committee (Approval number H-2018-0381).

### 2.4. Measures

Nutrition knowledge was measured using the Child Nutrition Knowledge Questionnaire-Australian version (CNK-AU), which consists of eight categories covering the following topics: ‘Healthy choices’; ‘AGHE serves’; ‘Balanced meals’; ‘Nutrient & food functions’; ‘Food categorisations’; ‘Food safety’; ‘Nutrition labels’ and ‘Food sources’. The CNK-AU was developed specifically for Australian children aged 9 to 12 years and was found to be a reliable and practical nutrition knowledge measurement tool in this age group [29].

At baseline, demographic questions about gender, age and year of school were asked at the start of the questionnaire. The likeability of VitaVillage was measured using seven questions, with rating scale response options ranging between 0 (negative) and 100 (positive). In addition, there was a multi-answer question (with an open answer option) question about what they would like to see added to the game in the future was included. Furthermore, participants were allowed to write comments and feedback within each likeability question and also at the end of the questionnaire. 

### 2.5. Statistical Analyses

Demographics and likeability scores were analysed using the descriptive statistics functions in IBM SPSS Statistic (version 25, IBM Corp., Armonk, NY, USA). Written feedback was qualitative data and reported by reading feedback responses and categorising responses.

The nutrition knowledge outcome variables were the differences between baseline (T1) and the end of the study (T2). Possible differences between the control and intervention group were investigated by an independent sample *t*-test. 

To test for differences in nutrition knowledge (categories and total scores) between the control and intervention at T2, independent *t*-tests were used. Additionally, paired *t*-tests were used to find changes from T1 to T2 within control participants. The same was done for intervention participants. A conservative approach to adjusting the *p*-values for multiple comparisons was taken to reduce the risk of Type 1 error by using a Bonferroni correction: *p* = 0.05/ number of outcomes (*n* = 8) = 0.0063 [30]. Thus, *p*-values of <0.0063 were considered significant.

## 3. Results

In total, 189 year 5 and 6 students were recruited. In the control group, 94 students could be matched between T1 and T2 and in the intervention group, 75 were matched. Other students could not be matched due to being absent at either T1 or T2. The sample’s characteristics can be found in Table 1.

The mean age in the control group was 10.9 (SD 0.8) and in the intervention group 10.9 (SD 0.7), with no statistical difference found. Girls comprised 53% of the control group and 41% in the intervention group.

The intervention group participants rated the VitaVillage game likeability 76.7 (SD 24.6). For their likeness of learning about nutrition via the game, they gave it the highest score 78.1 (SD 21.4) and the likeability of the goal of the game scored 73.7 (SD 26.6). The appearance of the game scored 69.0 (SD 25.3), the difficultness to play the game was on average 60.2 (SD 30.5), the difficultness of the quests and other content scored 55.9 (SD 25.3) and lastly, they rated on average 60.4 (SD 34.6) when asked how likely they were to tell their friends about the game. 

Table 2 shows the results of the multi-answer question, where participants could indicate which additions they would like to see included in the game. The most popular addition was having a choice of avatar (i.e., choosing/changing/adding to the player’s character in the game), with 73% of the participants selecting this option.

Changes in nutrition knowledge between T1 and T2 are presented in Table 3. None of the nutrition knowledge categories scores had significantly changed for the control group between T1 and T2. In the intervention group, participants improved their knowledge scores from T1 to T2 for ‘AGHE serves’ (ΔM0.9, SD1.7), ‘Balanced meals’ (ΔM0.3, SD1.2), ‘Food categorisations’ (ΔM0.6, SD2.4), ‘Food sources’ (ΔM0.3, SD1.1) and total nutrition knowledge scores (ΔM2.3, SD6.3). Moderate effects were found in the changes in nutrition knowledge scores from T1 to T2 between control and intervention for the categories ‘AGHE serves’, ‘Food categorisations’, ‘Nutrition labels’ and ‘Balanced meals’. The latter was significant (*p* = 0.006). The total nutrition knowledge score significantly improved between T1 and T2 for the intervention group. A moderate effect size was observed for improvement of the total nutrition knowledge score between control and intervention groups at T2. 

## 4. Discussion

Results of the current study show that the VitaVillage serious game was well received by the year 5 and 6 students.

### 4.1. Acceptability of VitaVillage

Children playing VitaVillage rated the overall game highly and most thought the game was an enjoyable way to learn about nutrition. However, many of them indicated that the game could be improved by adding a choice of avatar, multi-player options, maps, currency and saving functionality. 

A meta-analysis by DeSmet et al. on serious digital games for healthy lifestyle promotion noted that players seem to be more willing to learn through games, compared to traditional teaching methods, as long as the game’s challenge was feasible [31]. If the immersive features of a game are too difficult and increase cognitive load, it has been found that the educational content is poorly transferred [32]. In the current study, participants rated the difficulty of the game overall on average 60, and 55 for the difficulty level for the quests, on a scale of 0 to 100. This shows a good level of challenge in the game.

Many participants also indicated they would like to see multiplayer and customization options added to the game. This is consistent with the findings of Holzmann et al., who investigated children’s preferences and motives for nutrition education via digital gaming [4].

### 4.2. Improvement of Nutrition Knowledge

Compared to the control group, the intervention group’s overall nutrition knowledge was found to increase after playing VitaVillage. In addition, a positive effect was found for the knowledge categories ‘AGHE serves’, ‘Food categorisations’, ‘Nutrition labels’ and ‘Balanced meals’.

VitaVillage contains several quiz questions concerning the appropriate number of serves of fruit and vegetables to be consumed daily, which explains the medium effect-sized increase in AGHE serves knowledge. Similarly, a few balanced meal questions included in the game can explain the increase in that category’s score increase. However, as most quiz questions concerned knowledge about minerals, vitamins and their functions, it was expected the scores on ‘Nutrient & food functions’ would see a significant increase for the intervention group. Even though the increase in the mean score for the intervention group was higher compared to the control group, the difference was not statistically significant. Similar outcomes were reported by Hermans et al., who tested a serious nutrition education game which was played for a total of 60 minutes spread over two days [14]. No differences were found in nutrient function knowledge between intervention and control groups and the authors suggested increasing the game play duration and longer follow-ups might be the most effective in increasing children’s nutrition knowledge [14]. Therefore, even though some effects were found after playing VitaVillage in the current study, the exploratory nature of the study meant restrictions in measuring long-term retention of the knowledge, and future studies should test whether playing VitaVillage can have long-term benefits.

Furthermore, existing research has shown children’s memory to be strongly linked to emotions [33,34]. This might explain why the intervention group’s knowledge of nutrients and functions did not increase significantly; the story or information might not be emotionally engaging enough to motivate children to remember the facts. Further developments of the game should consider incorporating more emotionally engaging quests and features. 

One way of ensuring this is by incorporating the feedback provided by the participants in the current study, such as by adding multi-player settings, avatar options, a soundtrack, a VitaVillage currency or more varied quests. In addition, the developers of VitaVillage were restricted by the availability of graphical assets depicting food items, and adding food items such as ‘junk’ foods and snacks would add more options to the game (e.g., poor food choices result in negative town health points).

### 4.3. Feasibility

Future studies using revised versions of the game are warranted. Firstly, the game’s content should be expanded, allowing it to be played for a longer time or played more frequently. In the current study, VitaVillage was only played for 40 minutes in total. A study by Putnam et al. found that repeated exposure to their serious game was significantly correlated to learning nutritional information [35]. Therefore, greater improvement in nutrition knowledge might be accomplished when playing time is increased. Furthermore, considering it has been found that healthy eating behaviours can be promoted by nutrition education [36], a future VitaVillage study should test for any dietary pattern changes over time. The authors of the current study believe that an expansion of content will be needed to achieve behaviour change. The game will need a more concrete conceptualization of the behavioural skills required for making healthy food choices and a more theoretical framework should be developed to support healthy eating attitudes and behaviours.

VitaVillage was developed as a tool that could be used during nutrition education lessons in primary school, rather than serving as fully comprehensive nutrition education program. However, previous research has found that even though stand-alone games can be effective in increasing knowledge, games that are part of a multi-component program were more successful in changing health related behaviours [31]. Using VitaVillage as part of a more extensive nutrition education program could also provide opportunities to teach nutrition as a cross-curricular subject, which has been found to be appealing and feasible for teachers [9]. For example, incorporating a currency into the VitaVillage world could also teach basic economics while learning about nutrition. Many other opportunities for cross-curricular content in VitaVillage exist and could be adaptable to teacher’s highest needs.

### 4.4. Study Limitations

Several limitations to the current evaluation should be mentioned. Firstly, an important limitation to the study design was that VitaVillage was not tested against more traditional nutrition education lessons. This was due to this study being of an exploratory nature, plus there appears to be a lack of a standardised ‘traditional’ lessons in existence and the participating teachers indicated having time-constraints. In addition, these time constraints did not allow for the study design to include longer game play or more time between playing VitaVillage and additional follow-ups. Hence, it is unclear if nutrition knowledge or rather short-term memory was measured.

Furthermore, school class randomization was limited due to the importance of avoiding the risk of group contamination within the schools. All students in each study group being from only one school might have resulted in a lack of sample diversity. In addition, a further limitation of the study was that it was not possible to run a mixed model analysis, due to the limited school class information collected on the participants. Furthermore, the sample size was not sufficient to detect small effects as statistically significant.

In addition, no data was collected about the student’s previous nutrition education in each school. However, it is worth noting that no significant differences were found in baseline nutrition knowledge between study groups. 

Lastly, both schools recruited were independent schools, which implies on average a high socio-economic level of attending students. This might have had a positive effect on the student’s baseline nutrition knowledge [37] and familiarity with playing video games on tablet devices, potentially limiting generalisability.

## 5. Conclusions

In conclusion, results indicate that the students enjoyed playing VitaVillage and highlighted adaptions needed to make the game more engaging and immersive. Preliminary data shows some improvement in nutrition knowledge after playing VitaVillage for a short time. In the long term however, this might not be sufficient to sustain nutrition knowledge and change eating behaviours. In order to be a successful nutrition education tool, VitaVillage’s content and gameplay should be revised and extended to hold all aspects of nutrition and retain the player’s attention. In addition, by collecting more demographic data on the children, future studies will allow for more comprehensive statistical analyses when investigating nutrition knowledge scores.

It has been demonstrated that VitaVillage has potential for use in primary school classrooms and could be a useful resource to overcome barriers that teachers face when teaching about nutrition. The game could provide a low-cost, enjoyable, standardised nutrition education for all children in primary school. Furthermore, the value of playing VitaVillage for both children and teachers could be improved by integrating more curriculum subjects into the game. 

This exploratory study shows VitaVillage as a nutrition education tool is feasible and accepted by children. The results from this study can inform a future larger randomized control trial (RCT) study into the long-term effects of VitaVillage on children’s eating behaviour.

## Figures and Tables

**Table 1:** Descriptive statistics of matched (T1-T2) participants.

	Control Group			Intervention Group		
	Year 5	Year 6	Total	Year 5	Year 6	Total
Number of participants	46	48	94	34	41	75
Mean age (years)	10.3 (SD 0.5)	11.4 (SD 0.5)	10.9 (SD 0.8)	10.4 (SD 0.5)	11.41 (SD 0.6)	10.9 (SD 0.7)
Gender						
Male	22 (47.8%)	21 (43.8%)	43 (45.7%)	12 (35.3%)	22 (53.7%)	34 (45.3%)
Female	24 (52.2%)	26 (54.2%)	50 (53.2%)	22 (64.7%)	19 (46.3%)	41 (54.7%)
Other		1 (2.1%)	1 (1.1%)			

SD = Standard Deviation.

**Table 2:** Preferred additions to be made to the game, according to the participants.

What Should Be Added to the Game?	*n*	% of Participants
Choice of avatar	55	73%
Map of VitaVillage	49	65%
VitaVillage currency	49	65%
Multiplayer	46	61%
Save game	41	55%
Different quests (e.g., healthy lunchbox)	39	52%
Using day/night	38	51%
Theme music	37	49%
More interaction	37	49%
Other activities	35	47%

**Table 3:** Child Nutrition Knowledge Australian Questionnaire (CNK-AU) scores.

	T1 Control	T2 Control	Change in Knowledge Control Group (ΔT2_c_ − T1_c_^†^)	T1 Intervention	T2 Intervention	Change in Knowledge Intervention Group (ΔT2_I_ − T1_I_^†^)	ΔMT2_i_ − ΔMT2_c_^§^	
	M ± SD	M ± SD	ΔM^¶^ ± SD	M ± SD	M ± SD	ΔM ± SD	*p* Value	Cohen’s d
Healthy choices	7.9 ± 1.4	7.9 ± 1.3	0.0 ± 1.2	8.1 ± 1.2	7.9 ± 1.5	−0.2 ± 1.3	0.303	0.01
AGHE serves	2.2 ± 1.5	2.4 ± 1.3	0.2 ± 1.8	1.6 ± 0.2	2.6 ± 1.5	0.9 ± 1.7 *	0.016	0.38
Balanced meals	2.3 ± 1.0	2.1 ± 1.0	−0.2 ± 0.9	1.9 ± 1.0	2.2 ± 1.1	0.3 ± 1.2	0.006 *	0.45
Nutrient & food functions	9.3 ± 2.9	9.5 ± 3.3	0.2 ± 2.9	8.9 ± 2.6	9.4 ± 3.0	0.5 ± 3.7	0.626	0.08
Food categorisations	13.5 ± 2.7	13.8 ± 2.7	0.3 ± 1.6	13.9 ± 2.8	14.5 ± 2.7	0.6 ± 2.4 *	0.189	0.21
Food safety	7.1 ± 1.0	7.1 ± 0.9	0.0 ± 0.9	7.1 ± 1.2	7.1 ± 1.2	0.0 ± 0.9	0.655	0.07
Nutrition labels	1.1 ± 0.6	0.9 ± 0.6	−0.2 ± 0.4	1.0 ± 0.5	1.0 ± 0.5	−0.3 ± 0.5	0.282	0.17
Food sources	11.8 ± 1.3	12.1 ± 1.2	0.3 ± 1.3	11.5 ± 2.2	11.8 ± 2.0	0.3 ± 1.1	0.879	0.02
TOTAL **	55.2 ± 6.1	55.9 ± 7.0	0.7 ± 4.6	54.0 ± 7.6	56.3 ± 7.5	2.3 ± 6.3 *	0.035	0.35

^†^ ΔT2_c_ − T1_c_ = difference in mean scores at T2 compared to T1 for the control group; ΔT2_I_ − T1_I_ = difference in mean scores at T2 compared to T1 for the intervention group. ^§^ ΔMT2_i_ − ΔMT2_c_ = difference in mean score for control group at T2 compared to the difference in mean score for the intervention group at T2. M = mean; SD = Standard Deviation; ΔM^¶^ = Difference in mean. * Significant at *p* < 0.006. ** TOTAL = sum of all points.

## Data Availability

The data presented in this study are available on request from the corresponding author. The data are not publicly available due to privacy restrictions.
